# Two Distinct Clinical Patterns of Ibrutinib-to-Venetoclax Transition in Relapsed Chronic Lymphocytic Leukemia Patients

**DOI:** 10.3390/curroncol29040227

**Published:** 2022-04-15

**Authors:** Isacco Ferrarini, Francesca Gandini, Ettore Zapparoli, Antonella Rigo

**Affiliations:** 1 Section of Hematology, Department of Medicine, University of Verona, 37134 Verona, Italy; antonella.rigo@univr.it; 2 B-Cell Neoplasia Unit, Division of Experimental Oncology, Università Vita-Salute San Raffaele, 20132 Milan, Italy; gandini.francesca@hsr.it; 3 Centre for Omics Sciences, IRCCS Ospedale San Raffaele, 20132 Milan, Italy; zapparoli.ettore@unisr.it

**Keywords:** ibrutinib, venetoclax, chronic lymphocytic leukemia

## Abstract

Patients with chronic lymphocytic leukemia (CLL) relapsing on ibrutinib are often treated with the Bcl-2 inhibitor venetoclax. However, the transition from one agent to another poses some clinical challenges due to disease flares sometimes occurring right after ibrutinib interruption. Here, we describe three clinical vignettes highlighting two distinct patterns of ibrutinib-to-venetoclax transition. While patients following the favorable pattern transited to venetoclax without experiencing disease flare, the one patient who took the unfavorable path showed rapid disease rebound, with large-cell transformation occurring one week after ibrutinib interruption. A high burden of *BTK* and *PLCG2* mutations was found only in patients with the favorable transition pattern, suggesting that removing BTK inhibition might be particularly harmful if CLL cells are progressing through mechanisms external to the BTK axis.

## 1. Introduction

Chronic lymphocytic leukemia (CLL) is a low-grade B-cell malignancy characterized by the clonal expansion of CD19^+^CD5^+^ mature B lymphocytes. Despite its indolent clinical course, a significant fraction of patients require treatment due to progressive cytopenia or lymph node enlargement [1]. Nowadays, targeted therapies with BTK inhibitors and Bcl-2 antagonists are supplanting chemotherapy by virtue of their decreased toxicity and increased efficacy, especially in the context of high-risk biological subgroups [2,3,4]. The BTK inhibitor ibrutinib yields an overall response rate (ORR) of 91% and a median progression-free survival (PFS) of 44.1 months in the relapse/refractory setting [2]. The Bcl-2 antagonist venetoclax leads to an ORR of 75% with a median PFS of about 30 months as a single agent [5]. Although the optimal sequencing of novel agents for CLL is still a matter of clinical investigation, a large number of patients are now relapsing on ibrutinib, and facing the issue of how to optimally transit to the next-line therapy. Indeed, the transition from ibrutinib poses some clinical challenges. First, stopping the BTK inhibitor in patients who have developed resistance to it can rapidly increase the disease burden. Of note, during the course of clinical trials for ibrutinib-relapsing patients, amendments have been made to shorten the ibrutinib wash-out period prior to the next treatment, with the aim of reducing the risk of disease flare [6]. Second, if venetoclax treatment is being started, four to five weeks of therapy (the ramp-up period) might be needed to achieve disease control, thus exposing patients who had just interrupted ibrutinib to a dangerous time window [7]. Therefore, expert recommendations suggest to maintain ibrutinib during venetoclax ramp-up, with safe interruption once nodal and leukemic disease is under control [7]. However, this contrasts with the current regulatory European policy that does not allow the concomitant administration of ibrutinib and venetoclax. Such discrepancies create significant heterogeneity around the clinical management of this specific time frame within the CLL patient history. 

At our institution, we off-label overlap ibrutinib with venetoclax during the first two weeks of venetoclax ramp-up. We report here that this approach can generate two different transition patterns that might be driven by distinct mechanisms of ibrutinib resistance. 

## 2. Pattern A: The Good Transition

**Figure 1:**
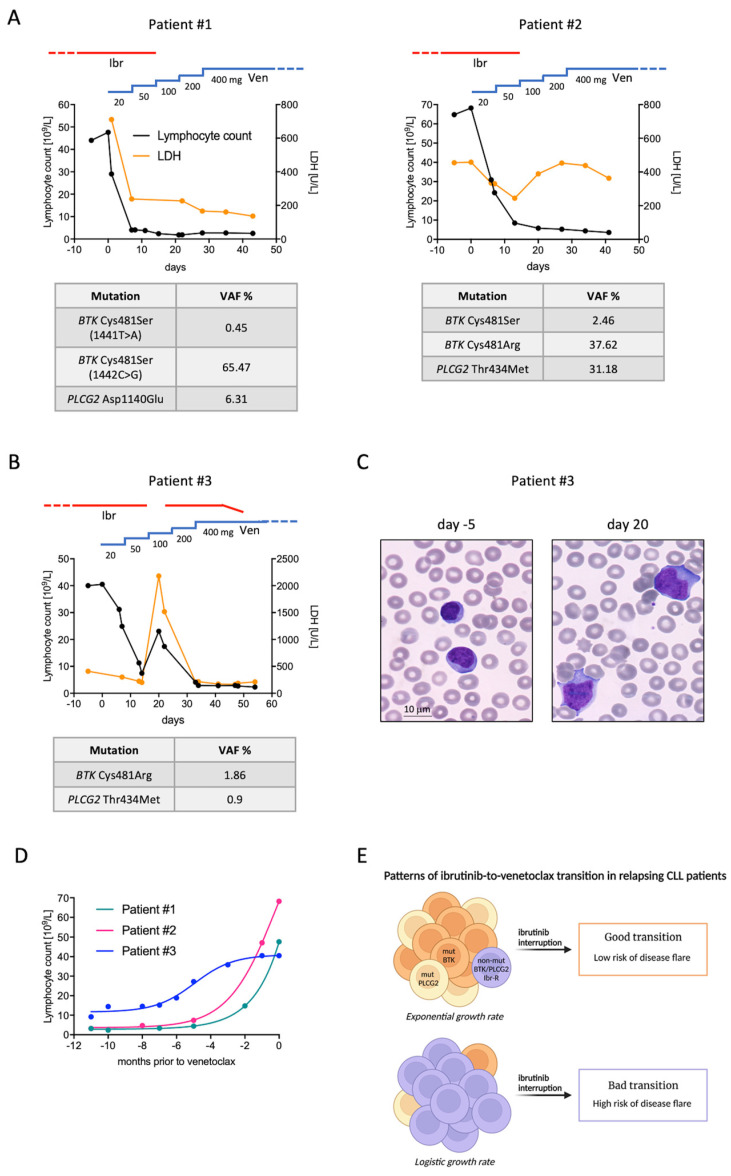
Clinical patterns of ibrutinib-to-venetoclax transition. (**A**) Lymphocyte count (black line), LDH values (orange line), and mutational analyses of patients undergoing the favorable transition pattern. (**B**) Lymphocyte count, LDH values, and mutational analysis of the patient undergoing the unfavorable transition pattern. (**C**) Peripheral blood smears (magnification 100×) of patient number #3 collected either before venetoclax initiation (while relapsing on ibrutinib, left panel) and after ibrutinib interruption (right panel). Small, mature lymphocytes were replaced by large cells with abundant basophilic cytoplasm and inconspicuous nucleoli. (**D**) Lymphocyte growth patterns of ibrutinib-relapsing patients described in this report. While patients #1 and #2 displayed an exponential growth rate, patient #3 showed a logistic growth pattern. (**E**) Working hypothesis resulting from ibrutinib-relapsing patients reported herein. Cases characterized by a high burden of *BTK* or *PLCG2* mutations display an exponential growth rate, but carry a relatively low risk of disease flare after ibrutinib interruption (good transition). Contrarily, those with a low level of *BTK* and *PLCG2* mutations show a more indolent relapse course and might continue to benefit from BTK inhibition. In these cases, ibrutinib interruption acutely restores the BTK signaling cascade, thus exposing patients to a higher risk of disease flare (bad transition). Ibr, ibrutinib; Ven, venetoclax; VAF, variant allele frequency; non-mut BTK/PLCG2 Ibr-R, ibrutinib-resistant cell without *BTK/PLCG2* mutations.

Patient #1. A 71-year-old man was diagnosed with CLL in 2013. Molecular and cytogenetic analyses showed wild-type *TP53*, unmutated *IGHV*, and del(11q). Flow cytometry was positive for ZAP-70 and CD38. Due to bulky adenopathy, he was treated with six cycles of bendamustine–rituximab (BR) in 2015, obtaining partial remission (PR). In May 2018, he experienced disease progression characterized by anemia due to extensive bone marrow infiltration, and bulky abdominal lymph nodes. He was started on ibrutinib at a standard dose with the achievement of PR that lasted until October 2021, when a second relapse occurred. His blood cell count showed rapidly evolving lymphocytosis (47,600/mmc) and thrombocytopenia (80,000/mmc), while CT scan documented the reappearance of multiple abdominal adenopathies (5 cm diameter) and splenomegaly (16 cm longitudinal axis). As shown in Figure 1A (left panel), venetoclax ramp-up was started without discontinuing ibrutinib for the first two weeks. After 14 days, lymphocyte count decreased to 2370/mmc and platelet count rose to 138,000/mmc. Ibrutinib was then withheld with no sign of disease flare over the following weeks. Molecular analysis of CLL cells collected prior to venetoclax initiation revealed two *BTK* Cys481Ser mutations (1441T>A and 1442G>C) and one *PLCG2* Asp1140Glu mutation with a variant allele frequency (VAF) of 0.85%, 65.47%, and 6.31%, respectively (see supplementary file for methodology).

Patient #2. A 72-year-old woman was diagnosed with CLL in 2014, characterized by unmutated *IGHV*, wild-type *TP53*, and trisomy 12. ZAP-70 and CD38 were not expressed. Due to progressive anemia, in 2017 she was treated with BR for six cycles, obtaining PR. This lasted until October 2019, when standard-dose ibrutinib was started due to anemia reappearance. PR was observed until October 2021, when she developed lymphocytosis with a doubling time of less than 6 months. Reassessment of biological features showed the emergence of *TP53* mutation. Venetoclax ramp-up was started and ibrutinib continued for the first two weeks. Lymphocyte count rapidly decreased to 5700/mmc and ibrutinib was stopped without clinical signs of disease flare, except for increasing lactate dehydrogenase (LDH) (Figure 1A, right panel). Molecular analysis of CLL cells collected prior to venetoclax initiation revealed two different *BTK* mutations, Cys481Ser and Cys481Arg, with a VAF of 2.46% and 37.62%, respectively. *PLCG2* Thr434Met mutation was also detected with a VAF of 31.18%. 

## 3. Pattern B: The Bad Transition

Patient #3. A 54-year-old man was diagnosed with CLL in 2010, characterized by unmutated *IGHV*, wild-type *TP53*, and no FISH abnormalities. Flow cytometry was negative for ZAP-70 and CD38. Cytogenetic analysis revealed a complex karyotype with del(6)(p21), add(8)(q24), and add(12)(p23). Due to bulky adenopathy and thrombocytopenia, he was treated with six cycles of FCR (fludarabine, cyclophosphamide, and rituximab) in 2011, obtaining PR. In 2014, he experienced symptomatic disease progression owing to bulky lymph node reappearance at his neck. Ibrutinib was started with the achievement of complete remission (CR) that lasted 6 years. In November 2020, lymphocyte count started rising, reaching up to 40,500/mmc in September 2021, accompanied by increased LDH (410 U/L, upper normal value 250 U/L). There was neither anemia nor thrombocytopenia, and an abdomen ultrasound showed mild splenomegaly (13.5 cm) with no lymph node enlargement. Because a further period of watchful waiting might have increased the risk of tumor lysis syndrome following venetoclax administration, the treating physician decided to start the third-line therapy with venetoclax prior to reaching the full clinical criteria for treatment initiation. Cytogenetics showed the acquisition of trisomy 1q in addition to the existing alterations, without *TP53* disruption. ibrutinib was maintained during the first two weeks of venetoclax ramp-up and withheld thereafter. The two weeks of combined treatment led to a marked decrease in lymphocyte count, which fell to 7400/mmc. LDH also decreased to 224 U/L. However, a dramatic disease flare occurred immediately after ibrutinib interruption (Figure 1B). Blood exams performed six days after ibrutinib withdrawal showed rebound lymphocytosis (23,050/mmc), thrombocytopenia (85,000/mmc), neutropenia (210/mmc), and markedly elevated LDH (2180/mmc). Peripheral blood smear documented large cells with inconspicuous nucleoli and abundant basophilic cytoplasm, mimicking leukemic Richter’s transformation (Figure 1C). Physical examination showed bilateral cervical and axillary lymph nodes that were not present before venetoclax initiation. The patient complained of night sweats and fatigue. Ibrutinib was then reintroduced at standard dose together with venetoclax, which was continued following the ramp-up schedule. Thirteen days later, lymphocyte count fell to 2900/mmc, neutrophils and platelets rose to 2090/mmc and 165,000/mmc, respectively, and LDH normalized (208 U/L). Symptoms and adenopathies completely resolved. Ibrutinib was gradually tapered and finally discontinued four weeks after the disease flare. The patient successfully completed venetoclax ramp-up and started rituximab as per clinical practice, with ongoing clinical response. Molecular analysis of CLL cells collected during ibrutinib progression (before venetoclax start) revealed that only a tiny fraction of the relapsing clone harbored *BTK* and *PLCG2* mutations. *BTK* Cys481Arg and *PLCG2* Thr434Met were detected with a VAF of 1.86% and 0.9%, respectively.

## 4. Discussion

These reports highlight two divergent clinical routes that might occur during the ibrutinib-to-venetoclax transition. Whilst patients who exhibit the favorable pattern (pattern A) can safely interrupt ibrutinib after the first two weeks of venetoclax ramp-up, those exhibiting the unfavorable one (pattern B) should stay on combined therapy for longer to avoid clinically relevant disease flare. It is noteworthy that the majority of CLL cells of patient #3, who showed pattern B, did not harbor *BTK* or *PLCG2* mutations and likely progressed through resistance mechanisms not involving the BTK signaling pathway. In such a cellular context, ibrutinib is no longer effective in preventing CLL growth (lymphocyte count reached up to 49,760/mmc while on ibrutinib), but is still effective in blocking oncogenic BTK signaling. Indeed, when ibrutinib was stopped the lymphocyte count immediately rose, along with the appearance of clinical signs of disease activity and aggressiveness. We speculate that the acute restoration of BTK signaling in cells that had already gained complementary pro-survival forces triggered the disease flare. This mechanism might underlie the CLL ‘hyperprogression’ sometimes observed among ibrutinib-relapsing patients waiting for next-line therapy. Moreover, such uncontrolled, yet transient, CLL proliferation might be partially responsible for the short PFS of ibrutinib-exposed patients treated with venetoclax as a single agent [6,8]. Paradoxically, the patient experiencing the disease flare displayed the most indolent progression on ibrutinib, characterized by a logistic lymphocyte growth pattern [9] (Figure 1D). By contrast, patient #1 and #2 showed an exponential increase in lymphocyte count during ibrutinib progression (Figure 1D), suggesting that autonomous BTK signaling confers proliferative advantage and leads to more rapid disease regrowth. 

On the road to therapy resistance, multiple subclonal mutations related or not to the BTK axis can emerge and evolve in parallel [10,11]. Based on these hypothesis-generating case reports (Figure 1E), we can envisage prolonging ibrutinib administration over the whole venetoclax ramp-up (and perhaps beyond) in patients not harboring meaningful sizes of *BTK* or *PLCG2* mutations. This pharmacological approach guarantees that BTK signaling, one of the driving forces of CLL, is controlled during disease debulking. Tailoring the transition strategy according to ibrutinib resistance mechanisms might eventually represent a novel way to improve the outcome of the subsequent venetoclax therapy.

## Data Availability

The data presented in this study are available on request from the corresponding author.

## Supplementary Materials

The following supporting information can be downloaded at: https://www.mdpi.com/article/10.3390/curroncol29040227/s1. Supplementary file include Sample collection, Library preparation and next generation sequencing and Bioinformatics analysis [12,13].
