# Supplementary material for: Two Distinct Clinical Patterns of Ibrutinib-to-Venetoclax Transition in Relapsed Chronic Lymphocytic Leukemia Patients
Source: Curr Oncol. 2022 Apr 15;29(4):2792–7. doi: 10.3390/curroncol29040227 (PMC9028984; doi:10.3390/curroncol29040227)
Supplement: Supplementary file 1 [file curroncol-29-00227-s001.zip › curroncol-1675324-supplementary.pdf]

Current Oncology

Supplementary Methods

Two Distinct Clinical Patterns of Ibrutinib-to-Venetoclax Transition in Relapsed Chronic Lymphocytic Leukemia Patients

Isacco Ferrarini, Francesca Gandini, Ettore Zapparoli and Antonella Rigo

Sample collection

B cells were purified from viable frozen peripheral blood mononuclear cells (PBMCs) using a positive-selection method (EasySep Human CD19 Positive Selection kit II, StemCell Technologies) for patient #1 and #2, and using a negative-selection immunodensity method (RosetteSep Human B Cells, StemCell Technologies) for patient #3. Genomic DNA (gDNA) was extracted using the QIAamp DNA Micro kit (Qiagen) and the concentration was determined using Qubit dsDNA HS assay (ThermoFisher).

Library preparation and next generation sequencing

A previously published custom Agilent HaloPlex High Sensitivity (HS) panel design [12,13] was modified using the Agilent SureDesign software (https://earray.chem.agilent.com/suredesign/) to additionally target the coding exons or hotspot regions of BTK and PLCG2. Libraries were prepared using 100 ng of high-quality gDNA input, following the manufacturer's instructions. Paired-end sequencing (150 bp reads) was performed on a NextSeq instrument (Illumina, USA). The average sequencing coverage for all exons of BTK and PLCG2 were 5,475x and 7,715x, respectively. The recurrently mutated BTK positions chrX:100611164 and chrX:100611165 (hg19) reached a mean sequencing-depth of 4,022x. The mutated PLCG2 positions 16:81934324 and 16:81973603 reached a mean sequencing-depth of 9,051x and 11,978x, respectively.

Bioinformatics analysis

Reads were aligned with bwa mem (v0.7.12) aligner on 1000 genomes Phase2 Reference Genome Sequence (hs37d5), based on NCBI GRCh37 assembly. HaloPlex Molecular Barcodes (MBC) were marked with LocatIt (v1.7) software provided by Agilent. Variants were called with Freebayes (v1.1.0) variant caller with the following parameters: -F 0.001 -k -w -V -a -J -K --report-genotype-likelihood-max -p 6 --min-base-quality 20 --strict-vcf.

Variants were then filtered for Shannon entropy of flanking sequence by 0.1 on both sides, for strand bias (SAF > 1 & SAR > 1), read balance (RPR > 1 & RPL > 1) and alternate observations (AO >= 2) using snpSift (v4.3t) software, and for indels pattern (bcftools filter -g 3 -G 1) using bcftools (v1.4) software. Annotation was done using databases such as dbSNP v146, COSMIC v73, Clinvar v20160802 and dbNSFP v2.9. Functional effect prediction was performed using SnpEff (v4.3i) software and only variants with MODERATE or HIGH impact were considered along with those having an effect involving a splice site.
